# Rapid and Cost‐Effective Digital Quantification of RNA Editing and Maturation in Organelle Transcripts by Oxford Nanopore Target‐Indexed‐PCR (TIP) Sequencing

**DOI:** 10.1002/pld3.70111

**Published:** 2025-10-20

**Authors:** Zhihua Hua

**Affiliations:** ^1^ Environmental and Plant Biology Department Ohio University Athens Ohio USA; ^2^ Interdisciplinary Program in Molecular and Cellular Biology Ohio University Athens Ohio USA

**Keywords:** chloroplast transcriptome, intron retention, *MORF2*, multiplexed amplicon sequencing, Oxford Nanopore sequencing, RNA editing

## Abstract

RNA editing and maturation are critical regulatory mechanisms in plant organelles, yet their quantification remains technically challenging. Traditional Sanger sequencing lacks sensitivity and reproducibility, whereas advanced next‐generation sequencing (NGS) approaches, such as rRNA‐depleted long non‐coding (lnc) RNA‐seq or targeted amplicon‐seq, involve high costs, complex workflows, and limited accessibility. To address these limitations, I developed a rapid and cost‐effective long‐read sequencing approach, termed Target‐Indexed‐PCR (TIP) sequencing, for digital quantification of RNA editing and intron retention events in targeted chloroplast transcripts. This method combines multiplexed high‐fidelity PCR amplification with Oxford Nanopore sequencing and custom in‐house Perl and Python scripts for streamlined data processing, including barcode‐based demultiplexing, strand reorientation, alignment to a pseudo‐genome, manual editing‐site inspection, and splicing variant identification. As a proof of concept, TIP sequencing was applied to *ndhB* and *ndhD* transcripts, two chloroplast *NAD*(*P*)*H dehydrogenase* genes with the highest number of known editing sites in *Arabidopsis thaliana* . These transcripts were analyzed both in an inducible CRISPR interference (iCRISPRi) system targeting *MORF2*, a key RNA‐editing factor, and in *MORF2‐YFP* transgenic lines with either overexpression or co‐suppression silencing. My findings revealed dose‐ and development‐dependent impacts of *MORF2* on C‐to‐U editing efficiency. Moreover, I identified an accumulation of intron‐retaining *ndhB* transcripts, specifically in Dex‐treated iCRISPRi lines and in both *MORF2‐YFP* overexpression and silencing rosette leaves, indicating impaired chloroplast splicing functions when *MORF2* expression is perturbed beyond an as‐yet‐undefined threshold. The platform achieves single‐molecule resolution, robust reproducibility, and high read coverage across biological replicates at a fraction of the cost of lncRNA‐seq. Collectively, this study establishes TIP sequencing as a versatile, scalable, and affordable tool for targeted post‐transcriptional analysis in plant organelles and expands our understanding of *MORF2*'s role in chloroplast RNA maturation. By overcoming key limitations of existing approaches, TIP sequencing enables routine, site‐specific quantification of post‐transcriptional regulation in organelles, including RNA editing and splicing, making it broadly accessible to researchers studying plastid biology, stress responses, and organelle–nucleus communication.

## 1 Introduction

The presence of multiple genomes in eukaryotic cells necessitates distinct regulatory mechanisms to coordinate gene expression between nuclear and organellar genomes, such as those found in mitochondria and chloroplasts in plant cells. This genomic compartmentalization has driven the evolution of intricate signaling networks essential for proper development and physiological adaptations through coordinated gene expression (Woodson and Chory 2008; Jarvis and Lopez‐Juez 2013). Among these regulatory mechanisms, RNA editing represents a distinctive post‐transcriptional modification predominantly observed in mitochondria and chloroplasts. Through site‐specific conversion primarily of cytidine (C) to uridine (U), RNA editing modifies transcripts to restore conserved codons, generates functional AUG start codons, eliminates premature stop codons, and influences RNA structure, splicing, and stability (Takenaka, Zehrmann, Verbitskiy, Hartel, et al. 2013; Small et al. 2020). These modifications are critical for organelle biogenesis and have been increasingly recognized as dynamic regulators of stress responses and developmental signaling (Hao et al. 2021; Hu et al. 2024; Mohamed et al. 2024).

RNA editing is particularly abundant in the organellar transcripts of land plants, with approximately 40 and 500 editing sites identified in chloroplast and mitochondrial RNAs, respectively, in *Arabidopsis thaliana* (Chateigner‐Boutin and Small 2007; Bentolila, Elliott, et al. 2008). These editing events often lead to amino acid substitutions crucial for the functionality of proteins involved in photosynthetic electron transport, ATP synthesis, and ribosome assembly (Sun et al. 2013; Takenaka, Zehrmann, Verbitskiy, Hartel, et al. 2013; Small et al. 2020). Recent studies further demonstrated dynamic regulation of editing efficiency in response to environmental stimuli such as temperature (Cui et al. 2019; Wu et al. 2025), light (Hu et al. 2025), and drought (Mohamed et al. 2024), underscoring RNA editing as an adaptable mechanism for environmental acclimation and signaling.

Despite its biological importance, quantitative analysis of RNA‐editing efficiency remains technically challenging. Multiple methodological strategies have been employed to measure RNA‐editing efficiency in plant organelles, each offering distinct advantages and limitations in terms of resolution, scalability, cost, and accessibility.

The traditional approach, Sanger sequencing of reverse transcription PCR (RT‐PCR) products, remains widely used for small‐scale validation of editing events (Takenaka, Zehrmann, Verbitskiy, Kugelmann, et al. 2012; Zhao et al. 2019; Tang et al. 2024; Wu et al. 2025). This method provides direct visualization of cytosine (C) to thymine (T) peak shifts in sequencing chromatograms and requires only basic laboratory infrastructure (Takenaka, Zehrmann, Verbitskiy, Kugelmann, et al. 2012). However, it is inherently low‐throughput and semi‐quantitative, and exhibits poor reproducibility, particularly when editing efficiencies are modest (< 20%) or high (> 80%), where chromatogram peak ratios are difficult to distinguish (Tang et al. 2024). Consequently, Sanger sequencing is not suitable for extensive comparative analyses across multiple genes, conditions, or biological replicates.

To improve throughput and quantification, strand‐ and transcript‐specific PCR sequencing (STS‐PCRseq) and targeted amplicon‐seq have been employed. These techniques involve the PCR amplification of specific organelle transcript fragments, followed by the preparation of a next‐generation sequencing (NGS) library and deep sequencing on Illumina platforms. STS‐PCRseq offers single‐nucleotide resolution and high sensitivity, facilitating comprehensive and high‐resolution profiling of RNA editing events in plastids and mitochondria of *A. thaliana* (Bentolila, Oh, et al. 2013). However, these targeted approaches still require comprehensive NGS workflows, including PCR amplicon fragmentation, adapter ligation, quality controls, and multiplexed sequencing, which collectively result in high experimental costs, extended turnaround times, and multiple labor‐intensive steps. Additionally, they depend heavily on access to sequencing facilities and PCR reproducibility.

In contrast, transcriptome‐wide RNA sequencing (RNA‐seq) has become increasingly popular for concurrently analyzing RNA editing, gene expression, and splicing, employing specialized bioinformatics pipelines such as ChloroSeq and REDItools (Malbert et al. 2018; Lo Giudice et al. 2020). However, conventional RNA‐seq typically utilizes poly(A) enrichment, preferentially capturing nuclear‐encoded transcripts while excluding non‐polyadenylated organellar RNAs. This underrepresentation severely limits statistical power for detecting RNA‐editing variations (Yapa et al. 2023).

To address this limitation, specialized rRNA‐depleted long non‐coding (lnc) RNA sequencing (lncRNA‐seq) protocols have been developed. These protocols employ ribosomal RNA depletion followed by reverse transcription using random primers, facilitating comprehensive and unbiased recovery of organellar transcripts (Xu et al. 2023; Tang et al. 2024). Although lncRNA‐seq significantly improves organellar transcriptome coverage, it remains the most costly method due to both the high price of rRNA depletion kits and the deep sequencing required for sufficient coverage of editing sites.

Collectively, the biological importance of organelle RNA editing and the limitations of current analytical methods underscore the critical need for a rapid, affordable, and scalable approach that delivers absolute, digital quantification of RNA‐editing events at defined sites without the high costs associated with extensive NGS pipelines. Here, I introduce Oxford Nanopore Target‐Indexed‐PCR (TIP) sequencing, a novel method specifically designed to provide fast turnaround (< 24 h), cost‐effective analysis ($5 per replicate per gene, lower than Sanger sequencing), high reproducibility, and digital quantification of RNA‐editing efficiency across multiple full‐length gene transcripts. This approach leverages Oxford Nanopore's long‐read sequencing platform to capture full‐length amplicons with single‐molecule precision.

TIP sequencing fills a critical methodological gap by enabling precise, absolute quantification of RNA‐editing events with exceptional sensitivity, rapid turnaround, robust reproducibility, and scalable throughput, while eliminating the need for complex bioinformatics analyses and costly deep sequencing workflows.

## 2 Results

### 2.1 Transcriptome Complexity Leads to Differential RT‐PCR Products of Chloroplast Genes

In one of our previous studies, we developed an inducible CRISPR interference (iCRISPRi) strategy for repressing the expression of *MULTIPLE ORGANELLAR RNA EDITING FACTOR 2* (*MORF2*) by co‐expressing a single guide RNA (sgRNA) targeted to the transcriptional start site (TSS) of *MORF2* and a Krüppel‐associated‐box (KRAB) domain fused to the carboxyl terminus of endonuclease‐deactivated Cas9 (dCas9‐KRAB). Because *morf2* null mutants are albino and developmentally lethal (Bisanz et al. 2003; Takenaka, Zehrmann, Verbitskiy, Kugelmann, et al. 2012; Yapa et al. 2023), the expression of *dCas9‐KRAB* was driven by a dexamethasone (Dex)‐inducible promoter (Aoyama and Chua 1997), whereas the sgRNA was constitutively expressed under the *Arabidopsis U6–26* promoter (Xing et al. 2014). Thus, only upon Dex treatment is dCas9‐KRAB expressed and guided by the sgRNA to the TSS of *MORF2*, enabling its transcriptional repression in a dose‐dependent manner. This inducible suppression allows the otherwise lethal *iCRISPRi‐MORF2* transgenic plants to remain viable (Yapa et al. 2023).

Given the known role of *MORF2* in regulating the chloroplast RNA‐editing process, our previous work also utilized both Sanger sequencing of RT‐PCR products and high‐throughput short‐read RNA‐Seq to verify reduced RNA‐editing efficiency in Dex‐treated, 7‐day‐old *iCRISPRi‐MORF2* seedlings. However, Sanger sequencing lacked sensitivity and reproducibility (Yapa et al. 2023). To further demonstrate this technical challenge, I examined the C‐to‐U editing efficiency at the first editing site of chloroplast *NAD*(*P*)*H dehydrogenase B* (*ndhB*) transcripts. I gel‐extracted and cleaned a 317‐bp RT‐PCR amplicon encompassing the site for Sanger sequencing analysis. Among three biological replicates obtained from the *iCRISPRi‐MORF2* line *P1–12*, only Replicate 3 yielded a relatively clean chromatogram, which allowed estimation of ~70% C‐to‐U editing efficiency based on the peak areas of C and T (arrowhead, Figure S1). In contrast, the chromatograms from the other two replicates showed multiple overlapping peaks at nearly every position, making it impossible to determine the correct sequence, editing site, or editing efficiency. This inconsistency highlights a persistent limitation of Sanger sequencing‐based RNA‐editing assays, which are not only insensitive and poorly reproducible but also laborious due to the need for separate RT‐PCR reactions and gel‐purification steps to isolate clean products for sequencing.

**FIGURE 1:**
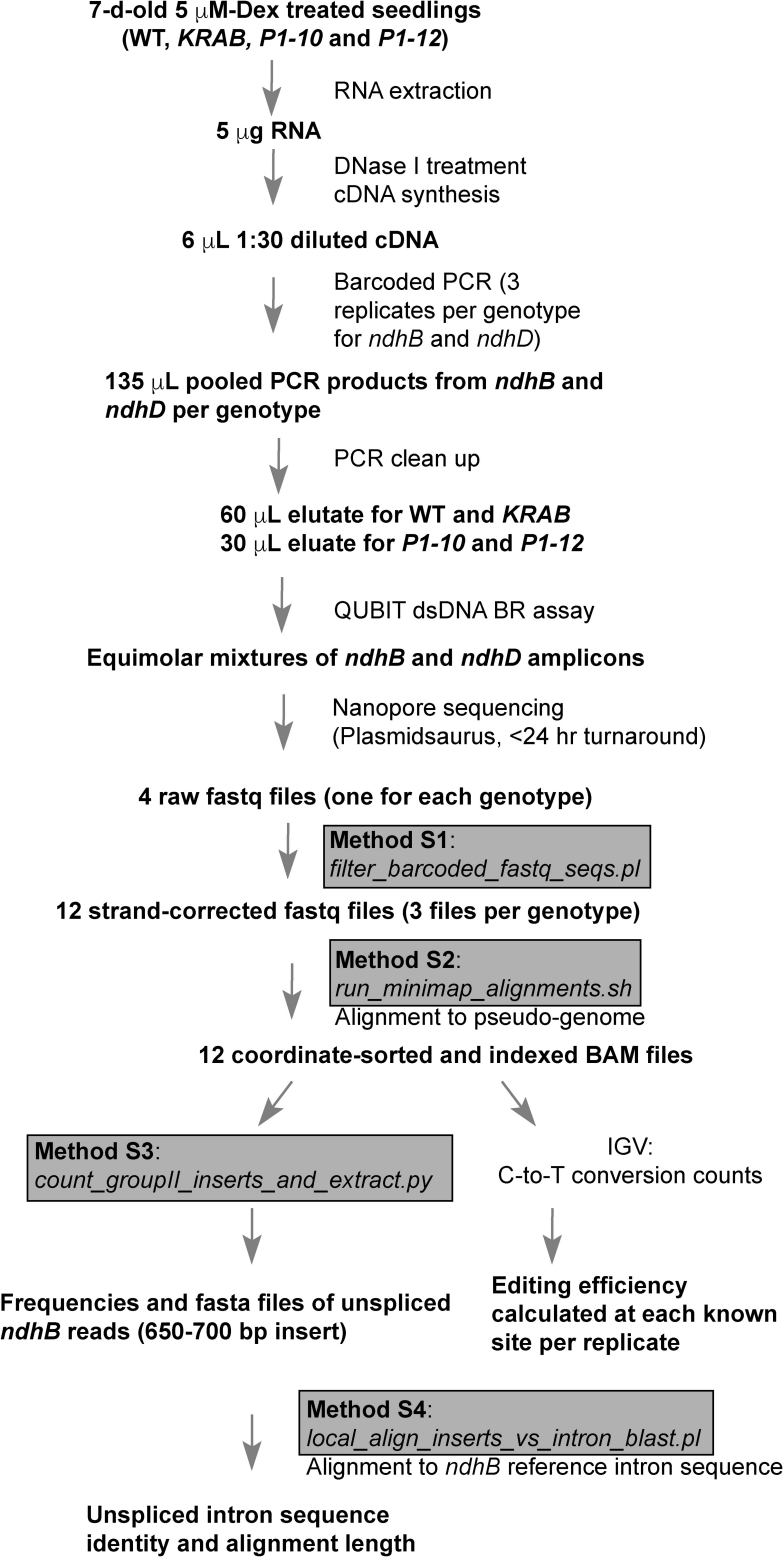
Workflow of TIP sequencing and data analysis for RNA‐editing efficiency and intron retention. A schematic diagram illustrating the experimental and analytical steps of the TIP sequencing platform. Custom open‐source bioinformatics scripts (Methods [Link], [Link], [Link]–S4) were developed in‐house to streamline the workflow for barcode demultiplexing, strand correction, pseudo‐genome alignment, editing‐site quantification, and intron‐retention analysis.

To overcome the limitations of Sanger sequencing‐based RNA‐editing analysis while avoiding the high cost associated with RNA‐seq–based approaches (see Section 1), I revisited the same *iCRISPRi‐MORF2* lines to develop a nanopore‐based long‐read sequencing platform, termed TIP sequencing, paired with custom bioinformatics tools for accurate and cost‐effective analysis of chloroplast RNA editing and transcript maturation (Figure 1). I focused on *ndhB* and *ndhD*, two chloroplast *NAD*(*P*)*H dehydrogenase* genes that harbor the highest number of C‐to‐U editing sites among chloroplast transcripts (nine and five, respectively; Bentolila, Oh, et al. 2013). Notably, *ndhB* also contains a group II intron (Ostheimer et al. 2003), enabling additional investigation of *MORF2*'s potential role in RNA maturation and splicing, given its known interactions with multiple Pentatricopeptide Repeat (PPR) proteins that are implicated in chloroplast RNA splicing (Takenaka, Zehrmann, Verbitskiy, Kugelmann, et al. 2012; Bayer‐Csaszar et al. 2017; Small et al. 2023; Zhang et al. 2023) and in maturation of the plastid ribosomal RNAs (Bisanz et al. 2003).

**FIGURE 2:**
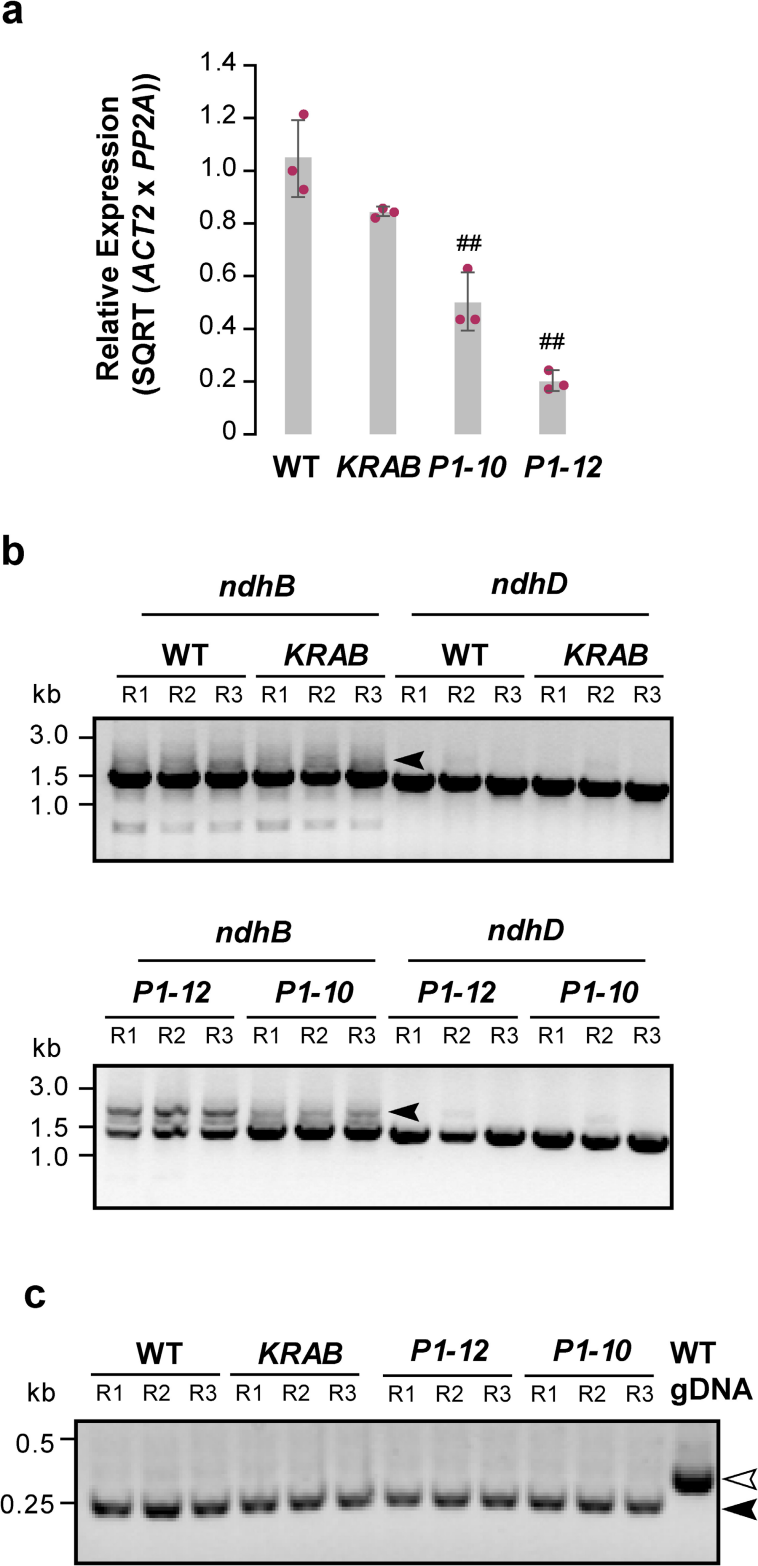
*MORF2* suppression by iCRISPRi alters *ndhB* transcript profiles. (a) Quantitative PCR analysis showing suppression of *MORF2* transcript levels in 7‐day‐old seedlings treated with 5‐μM Dex. Expression values are normalized to one WT replicate. *ACT2* and *PP2A* were used as internal reference genes. Bars represent the mean ± SD of three biological replicates, each measured in technical duplicate. Individual data points (maroon dots) show biological replicates. ## denote statistically significant differences from WT (*p* < 0.01; Student's *t* test, one‐tailed with unequal variance). (b) Gel electrophoresis images of RT‐PCR products for *ndhB* and *ndhD* amplified from the same cDNAs used in Subpart (a). R1–R3 indicate three biological replicates. Forward primers were barcoded for multiplexing. Five microliters of each 50‐μL PCR reaction were loaded on a 1% agarose gel stained with ethidium bromide. The arrowhead marks a putative unspliced *ndhB* amplicon. (c) Gel images of RT‐PCR products of *ACT2* from the same cDNAs as in Subpart (b), along with a genomic PCR control using *ACT2* primers on DNA from 7‐day‐old WT seedlings. Open and solid arrowheads indicate genomic (containing a 78‐bp intron) and properly spliced cDNA products, respectively.

Using quantitative PCR (qPCR), I verified that *MORF2* transcript levels were substantially reduced by 5‐μM Dex to 50.2% ± 10.8% in *P1–10* and 20.1% ± 3.9% in *P1–12* relative to wild type (WT) (mean ± SD). In contrast, *KRAB* plants expressing only Dex‐inducible *dCas9‐KRAB* retained 84.3% ± 3.9% of WT *MORF2* transcript levels, which were not statistically different from WT (Figure 2a). This gradient of *MORF2* repression enabled quantitative comparison of its dose‐dependent effect on *ndhB* and *ndhD* transcripts.

To obtain long cDNA products for nanopore sequencing, I synthesized first‐strand cDNA using a combination of oligo (dT)25 and random hexamer primers, followed by high‐fidelity PCR amplification of *ndhB* and *ndhD* cDNAs. The resulting amplicons displayed genotype‐dependent differences in both yield and complexity. Robust PCR products were observed for both *ndhB* and *ndhD* in WT and *KRAB*. In contrast, *ndhB* amplicons were moderately and strongly reduced in *P1–10* and *P1–12*, respectively, whereas *ndhD* products were only mildly affected in the same two genotypes, suggesting gene‐specific sensitivity of transcript processing to *MORF2* suppression (Figure 2b).

Notably, in addition to the expected full‐length *ndhB* band (~1.4 kb), larger amplicons appeared specifically in Dex‐treated *P1–10* and *P1–12* lines, with the signal particularly pronounced in *P1–12*. These upper bands likely correspond to intron‐retaining, premature mRNA isoforms (arrowheads, Figure 2b). To rule out the possibility of genomic DNA (gDNA) contamination, I compared the PCR products of *ACT2* from the cDNAs with those from WT gDNA. All produced a clean band, but the WT gDNA yielded a longer amplicon than the cDNAs, confirming that the cDNAs were derived from properly spliced transcripts (Figure 2c).

These complex banding patterns precluded standard Sanger sequencing for editing analysis, as clean chromatograms could only be obtained from single, specific PCR products. In our previous studies, we had to design internal primers to amplify smaller *ndhB* fragments, followed by gel extraction; however, this process was time consuming, poorly reproducible, and yielded inconsistent sequencing results (Yapa et al. 2023).

### 2.2 High Read Coverage of *ndhB* and *ndhD* Transcripts From Nanopore Long‐Read Sequencing

Because nanopore long‐read sequencing analyzes DNA strands at the single‐molecule level, I indexed the *ndhB* and *ndhD* PCR amplicons from each biological replicate using their respective forward primers and pooled the three replicates, 135 μL in total (each with 45 μL per replicate from an initial 50 μL PCR reaction), without performing an additional gel extraction step. Based on the stronger band intensity of *ndhB* and *ndhD* PCR products observed in WT and *KRAB* than in *P1–10* and *P1–12* from DNA gel electrophoresis (Figure 2b), I eluted the cleaned DNA in 60 μL for WT and *KRAB*, and 30 μL for *P1–10* and *P1–12*. Using the Qubit double‐stranded (ds)DNA Broad Range (BR) assay, I confirmed that each cleaned product exceeded 50 ng/μL in concentration.

To ensure an unbiased representation of *ndhB* and *ndhD* PCR amplicons in each final nanopore sequencing library, I prepared equimolar mixtures of *ndhB* and *ndhD* PCR products from each genotype. As expected, the resulting sequencing output yielded comparable read counts for *ndhB* and *ndhD* within each genotype, with totals ranging from 891 to 1300 reads (Table S1; Appendix S1).

Interestingly, the barcode sequence appeared to influence the number of reads obtained in TIP sequencing. Among the three barcode samples, ATGCTAGC (Replicate 1) produced the highest number of reads, followed by CGTACGTA (Replicate 2) and TACGATCG (Replicate 3) (Table S2), suggesting that barcode sequence may affect read recovery efficiency in nanopore sequencing.

### 2.3 Digital Quantification Confirmed *MORF2* Dose‐Dependent RNA‐Editing Changes

The high read coverage from each TIP sequencing run, ranging from 122 to 636 reads per replicate (Table S2), provided strong statistical power to digitally quantify RNA‐editing efficiency across genotypes. Using an in‐house Perl script that incorporates three BioPerl modules, *Bio::SeqIO*, *Bio::Seq*, and *Bio::Seq::Quality* (Figure 1; Method S1), I processed the sequence and quality data in each raw FASTQ file (Appendix S1) into strand‐corrected versions. Each read sequence and its corresponding quality score were oriented to match the sense strand of *ndhB* and *ndhD*. Based on the presence of a specific barcode within each sequence, I reconstructed three strand‐corrected FASTQ files from each TIP sequencing run for the *ndhB* and *ndhD* amplicons, with each file representing the sequencing result of one biological replicate per genotype, as indicated in Figure 2b.

I then aligned the strand‐corrected reads to a pseudo‐genome (Figure 1; Method S2; Appendix S2), which is composed of six synthetic chromosomes, each corresponding to a barcoded version of the *ndhB* or *ndhD* reference cDNA sequences. This yielded three binary alignment map (BAM) and BAM index (BAI) files for the three biological replicates of each genotype. Because the positions of the known editing sites were predefined, I manually quantified the number of cytosine (C) and thymine (T) bases at each site across genotypes by visualizing the indexed BAM files using the Integrative Genomics Viewer (IGV) browser (Table 1; Robinson et al. 2011).

**TABLE 1:** Counts of cytidine (C) and thymidine (T) nucleotides at known RNA‐editing site in *ndhB* and *ndhD* RT‐PCR products from four genotypes.

Site	Position^a^	Surrounding nucleotides	WT						*KRAB*						*P1_10*						*P1_12*					
			R1		R2		R3		R1		R2		R3		R1		R2		R3		R1		R2		R3	
			C	T	C	T	C	T	C	T	C	T	C	T	C	T	C	T	C	T	C	T	C	T	C	T
*ndhB1*	149	TTCAA	10	312	15	334	7	146	21	357	11	296	1	160	15	355	3	218	4	123	52	224	25	151	9	79
*ndhB2*	467	TCCAG	8	308	17	321	3	151	16	337	16	278	4	157	14	336	14	202	14	110	52	210	20	150	7	74
*ndhB3*	586	TTCAT	10	306	13	318	1	152	17	335	8	283	2	159	15	330	11	204	4	115	26	225	11	158	2	78
*ndhB4*	746	TTCTC	7	272	8	282	3	135	11	307	5	260	0	141	10	301	4	182	4	99	28	198	13	138	4	67
*ndhB5*	830	TTCAG	3	295	2	319	2	142	7	327	6	271	3	146	8	321	10	189	1	112	16	206	7	135	5	70
*ndhB6*	836	TTCAG	11	285	15	303	6	137	15	320	15	257	9	140	25	304	11	189	18	94	39	181	26	117	15	60
*ndhB7*	872	CTCAT	5	290	9	311	3	142	9	328	12	258	8	138	11	317	6	189	3	107	19	201	7	132	6	67
*ndhB8*	1255	TCCAT	11	307	10	310	1	141	9	344	10	291	3	140	16	330	8	202	1	113	33	232	21	140	7	70
*ndhB9*	1481	ACCAG	16	252	11	232	10	106	17	276	12	219	3	109	23	273	12	164	10	90	37	172	20	110	15	50
*ndhD1*	2	CACGA	279	287	148	150	132	111	235	243	187	157	133	120	307	249	181	138	153	81	362	56	194	42	140	22
*ndhD2*	383	ATCAT	10	498	10	265	6	224	12	436	14	313	7	224	27	478	11	294	13	200	30	355	9	224	7	139
*ndhD3*	674	ATCAC	41	430	19	244	11	201	27	405	15	290	14	207	27	446	9	280	11	200	23	335	15	202	9	129
*ndhD4*	878	TTCAA	62	397	39	215	36	168	51	363	38	258	34	183	97	367	39	250	47	154	110	233	63	145	34	102
*ndhD5*	887	TCCCG	52	403	42	216	32	171	49	362	32	257	27	187	54	402	25	263	37	163	51	291	36	173	24	107

^a^ Editing site positions are referenced to the first nucleotide of each gene's coding sequence. R1–R3 indicate three independent biological replicates.

**FIGURE 3:**
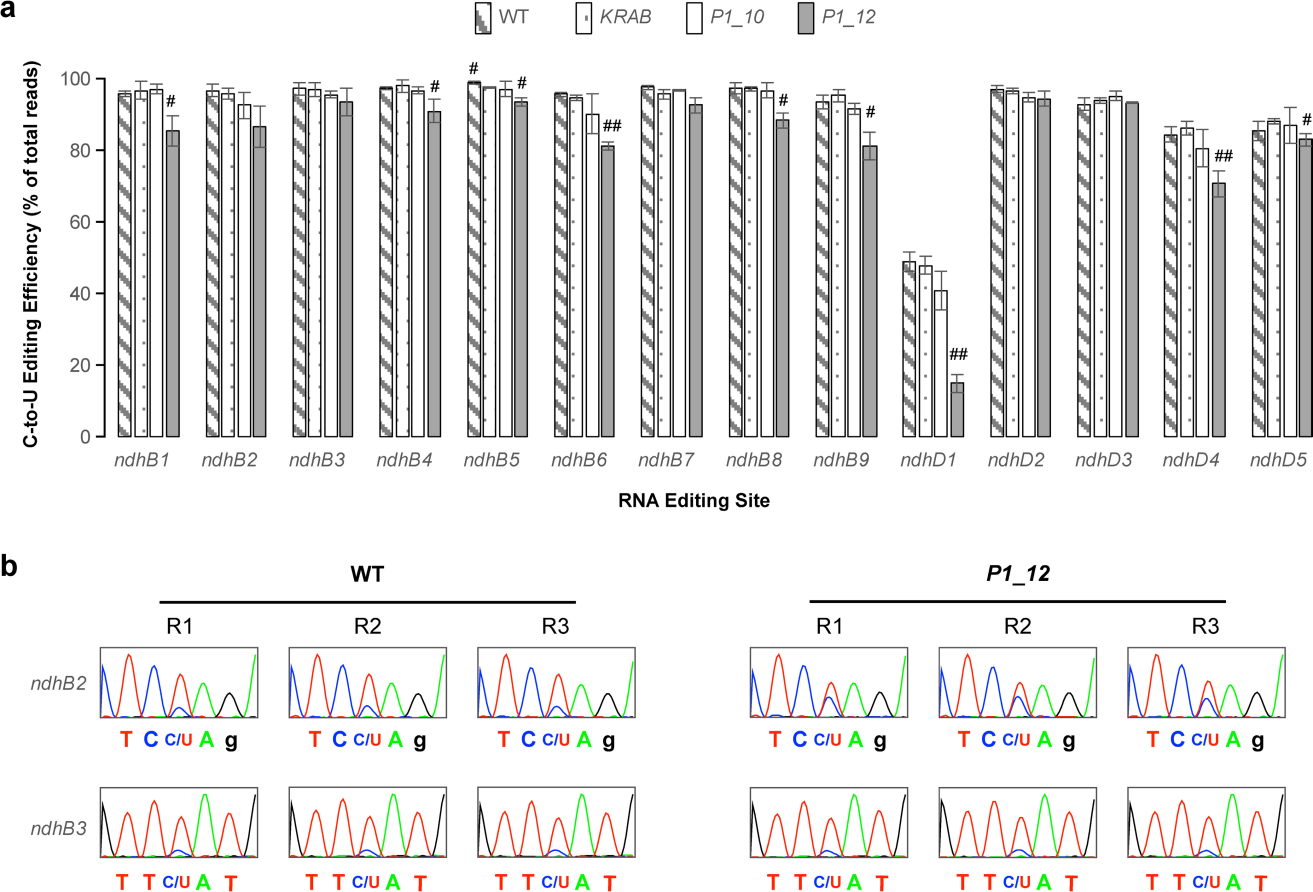
Reduced *MORF2* transcription impairs RNA editing in *ndhB* and *ndhD* transcripts. (a) TIP sequencing reveals a dose (threshold)‐dependent effect of *MORF2* on C‐to‐U RNA‐editing efficiencies in *ndhB* and *ndhD* transcripts. The editing efficiencies were digitally quantified from Oxford Nanopore TIP sequencing of RT‐PCR amplicons, as shown in Figure 2b. Data represent the mean ± SD of three biological replicates (see Table 1). Refer to Table 1 for the positions and surrounding nucleotide contexts of each RNA‐editing site. Statistically significant differences from *KRAB* are indicated (#*p* < 0.05, ##*p* < 0.01; Student's *t* test, two‐tailed with unequal variance). (b) Sanger sequencing chromatograms of RT‐PCR products spanning the *ndhB2* and *ndhB3* sites in three biological replicates of WT and *P1_12*. Partial C‐to‐U editing at each site is indicated by overlapping peaks (C/U).

In total, I observed 65 to 556 valid reads per editing site across all sequencing files (median count = 217), providing strong statistical power to quantify C‐to‐U editing efficiency (Table 1). TIP sequencing results revealed statistically significant reductions in editing efficiency at six out of nine *ndhB* and two out of five *ndhD* editing sites in *P1–12* compared with WT (Figure 3; *p* < 0.05, Student's *t* test, two‐tailed with unequal variance). In contrast, no significant differences were found in *P1–10*, suggesting that RNA‐editing defects, at least in *ndhB* and *ndhD*, occur in a *MORF2* dose‐dependent manner. These findings are consistent with our prior Sanger sequencing results at five *ndhB* editing sites, which also demonstrated a dose (threshold)‐dependent effect of *MORF2* on RNA editing (Yapa et al. 2023).

However, our earlier results indicated more severe reductions in editing efficiency in *P1–12*, with editing efficiencies of ~20% and ~10% at the *ndhB2* and *ndhB3* sites, respectively, based on Sanger sequencing of poly(A)‐derived RT‐PCR products (Yapa et al. 2023). In contrast, TIP‐sequencing in this study detected 86.6% ± 5.8% and 93.5% ± 3.9% editing efficiency at *ndhB2* and *ndhB3*, respectively (Figure 3a). To resolve this discrepancy, I used the new set of cDNAs and conducted Sanger sequencing of the RT‐PCR products spanning the *ndhB2* and *ndhB3* sites. Chromatogram peak area analysis from three biological replicates revealed editing efficiencies of ~80% and ~85% in WT, and ~67% and ~85% in *P1–12* at *ndhB2* and *ndhB3*, respectively (Figure 3b), which are closely matching the TIP‐sequencing results (Figure 3a).

The differences observed in our earlier study likely arose from (1) the use of poly(A)‐primed cDNA synthesis, which may introduce bias when analyzing chloroplast transcripts, (2) analog estimation of editing levels from low‐resolution chromatograms, and (3) reliance on a single biological replicate. In contrast, the agreement between the current Sanger and TIP‐sequencing results at both *ndhB2* and *ndhB3* sites reinforces the accuracy and utility of TIP‐sequencing for digital quantification of RNA‐editing efficiency.

Interestingly, I also detected a modest but statistically significant reduction in editing efficiency at *ndhB5* in *KRAB* compared with WT (Figure 3a, 99.0% in WT vs. 97.9% in *KRAB*; *p* = 0.035, Student's *t* test, two‐tailed with unequal variance), suggesting a possible off‐target or stress‐related effect of *dCas9‐KRAB* expression. Such minor but statistically significant differences could not be detected by Sanger sequencing (Yapa et al. 2023; Tang et al. 2024), further highlighting the increased sensitivity of our approach.

### 2.4 A High Frequency of Unprocessed Premature *ndhB* Transcripts in *P1–10* and *P1–12* Upon Dex Treatment

The presence of an additional PCR product band with a molecular weight higher than the expected 1405‐bp *ndhB* transcript (Figure 2b, indicated with an arrowhead), and its apparent correlation with the extent of *MORF2* suppression, suggests that this band represents a precursor *ndhB* transcript retaining a 685‐bp group II intron (TAIR, https://www.arabidopsis.org). To test this hypothesis, I included rigorous DNase treatment during RNA isolation and prior to cDNA synthesis and confirmed the absence of genomic DNA contamination by demonstrating no RT‐PCR amplification of the intron‐containing fragment of nuclear gene *ACT2* in any of the 12 cDNA samples (Figure 2c). These results support the hypothesis that the larger *ndhB* amplicons originated from unspliced premature RNAs rather than from genomic DNA.

To determine the nature of these long‐insert transcripts, I developed a Python script to extract reads containing insertions ranging from 650 to 700 bp from all three replicates of each genotype (Figure 1; Method S3). Using BLASTN pairwise sequence alignment (Figure 1; Method S4), I identified that these inserts shared 98.9% ± 2.1% sequence identities and an average alignment length of 685 ± 5 bp with the reference *ndhB* group II intron (Table S3), indicating that they are indeed unspliced intron sequences present in the premature *ndhB* transcripts.

I next quantified the frequency of these unprocessed *ndhB* transcripts in each genotype. Both *P1–10* and *P1–12 iCRISPRi‐MORF2* lines treated with 5‐μM Dex exhibited significantly higher frequencies of premature *ndhB* transcripts compared with WT (Table 2), consistent with the more intense high‐molecular‐weight bands observed in their RT‐PCR products (Figure 2b). Although the *KRAB* control line also showed a slightly elevated mean frequency compared with WT, this difference was not statistically significant (Table 2).

**TABLE 2:** Frequency of unspliced *ndhB* transcripts across seven genotypes or tissues.

	WT			*KRAB*			*P1_10*			*P1–12*			WT_RL			*g_RL*			*sil_RL*		
Replicates	R1	R2	R3	R1	R2	R3	R1	R2	R3	R1	R2	R3	R1	R2	R3	R1	R2	R3	R1	R2	R3
Unspliced reads	4	4	4	10	15	5	21	8	4	40	27	9	2	1	1	9	9	4	34	14	6
Aligned reads	531	439	196	614	471	215	585	337	186	501	330	122	594	270	96	756	310	136	787	233	70
Frequency (mean ± SD)^a^	1.2 ± 0.7			2.4 ± 0.8			2.7 ± 0.8*			7.8 ± 0.4**			0.6 ± 0.4			2.3 ± 1.0***			6.3 ± 2.1***		

Abbreviations: *g*, green non‐silencing; RL, rosette leaf; *sil*, silencing.

^a^ Frequency values represent the mean ± SD percentage of reads containing a 650–700 bp insert relative to total aligned reads per replicate. R1–R3 indicate three independent biological replicates as in Table 1.

* *p* < 0.05 (Students' *t*‐test against WT, one‐tailed, unequal variance).

** *p* < 0.01 (Students' *t* test against WT, one‐tailed, unequal variance).

*** *p* < 0.05 (Student's *t* test against WT_RL, one‐tailed, unequal variance).

### 2.5 Broad Applications of TIP Sequencing in Defining RNA‐Editing and Splicing Variations

To expand the scope of TIP sequencing application, I applied it to examine RNA‐editing changes in *ndhB* and *ndhD* and splicing variations of *ndhB* in Arabidopsis across developmental changes as well as in response to both up‐ and downregulation of *MORF2* in adult rosette leaves (RL) in *Yellow Fluorescent Protein* (*YFP*) tagged *35S:MORF2‐YFP* overexpression transgenic plants (Yapa et al. 2023).

**FIGURE 4:**
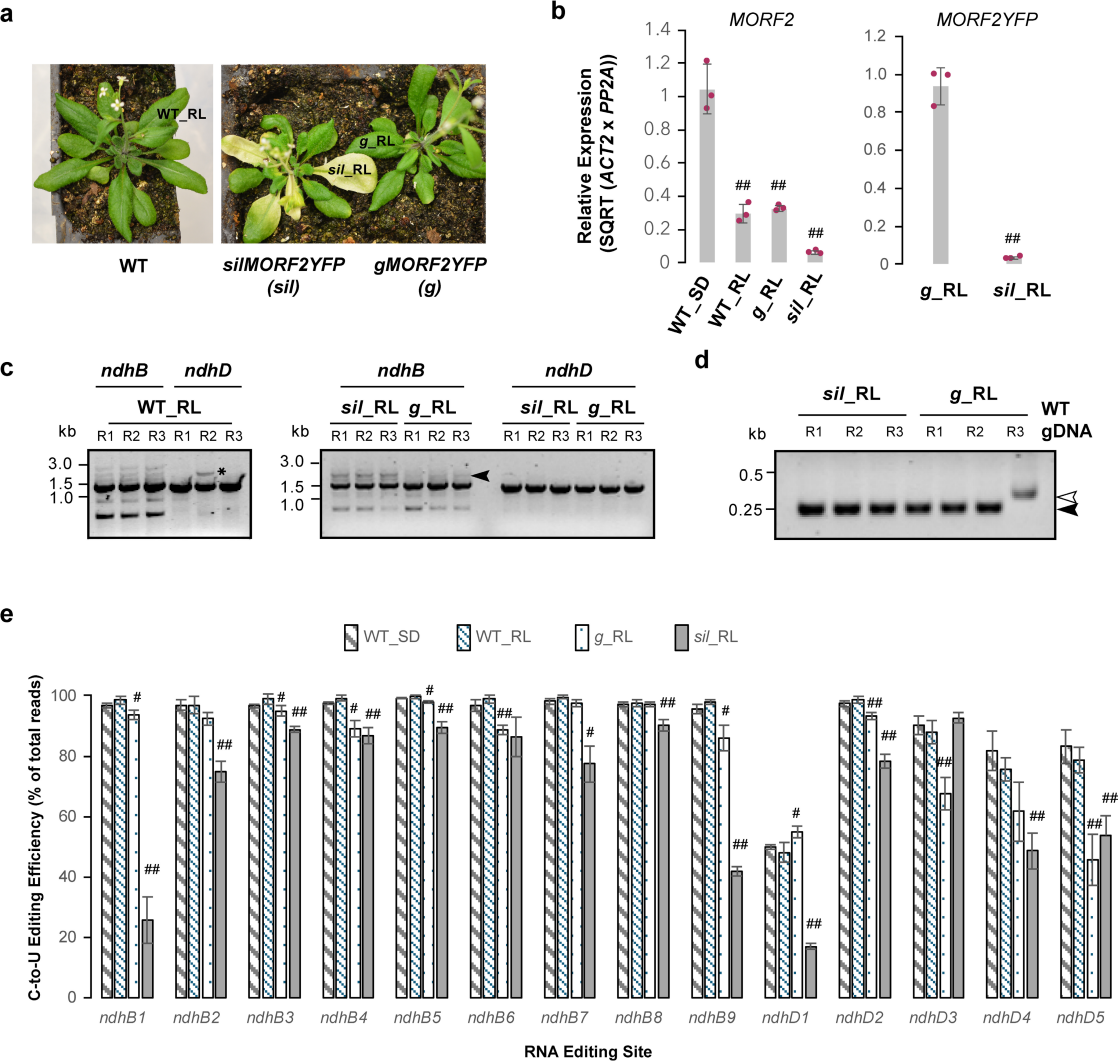
Comparative RNA editing efficiencies in *ndhB* and *ndhD* across developmental stage and *MORF2* perturbation. (a) Growth phenotypes of 4‐week‐old plants grown on soil. Adult rosette leaves (RLs) selected for RNA extraction and editing analysis are marked. (b) Quantitative PCR analysis showing developmental downregulation or transgene co‐suppression of the endogenous *MORF2* and *MORF2‐YFP* transgenes in RLs from the indicated genotypes. Expression values are normalized to one WT_SD replicate (left panel) or one *g*_RL replicate (right panel). *ACT2* and *PP2A* were used as reference genes. Individual data points and statistical analysis are as described in Figure 2a. Double hash tags (##) indicate statistically significant differences from WT_SD (left panel) or *g*_RL (right panel) (*p* < 0.01; Student's *t* test, one‐tailed with unequal variance). (c) Gel electrophoresis of RT‐PCR products for *ndhB* and *ndhD* amplified from the same cDNAs used in Subpart (b). R1–R3 indicate three biological replicates. Primer indexing and gel conditions are as described in Figure 2b. The arrowhead marks a putative unspliced *ndhB* amplicon, and the asterisk indicate a non‐specific amplicon. (d) RT‐PCR of *ACT2* confirming the absence of genomic DNA contamination in the cDNA samples used in Subparts (b) and (c). Solid and open arrowheads indicate spliced cDNA and genomic DNA products (containing a 78‐bp intron), respectively. (e) TIP sequencing reveals a threshold‐dependent effect of *MORF2* on C‐to‐U RNA‐editing efficiencies in *ndhB* and *ndhD* transcripts. The editing efficiencies were digitally quantified from Oxford Nanopore TIP sequencing of RT‐PCR amplicons in Subpart (c). RNA editing sites and data analysis are as described in Figure 3a. Statistically significant differences from WT_SD are indicated (# or *p < 0.05, ##*p* < 0.01; Student's *t* test, two‐tailed with unequal variance).

The *MORF2‐YFP* transgenic plants were generated in our previous studies and show spontaneous co‐suppression of both the transgene and endogenous *MORF2* across individuals and tissues, resulting in a chlorotic phenotype (Figure 4a). To verify the expression changes of the endogenous *MORF2* gene across developmental stages/tissues and the *MORF2‐YFP* transgene between silencing (*sil*) and non‐silencing green (*g*) RLs, I conducted qPCR analysis using primers specific to the endogenous *MORF2* 3′‐untranslated region and *YFP*. I unexpectedly discovered that the endogenous *MORF2* expression is developmentally regulated, with expression in adult RLs from 4‐week‐old plants reduced to 29.6% ± 5.6% of that in 7‐day‐old seedlings (Figure 4b). As expected, this developmental reduction did not result in a significant difference in the adult RLs between 4‐week‐old WT and *gMORF2‐YFP* plants, whereas the expression of *MORF2* was dramatically reduced to 6.1% ± 1.0% of 7‐day‐old seedlings in *sil_*RLs (Figure 4b). Consistently, the expression of the *MORF2‐YFP* transgene was suppressed to 3.2% ± 0.7% in *sil_*RLs compared with *g_*RLs (Figure 4b), confirming co‐suppression‐mediated downregulation of both endogenous *MORF2* and *MORF2‐YFP* in *sil_*RLs.

I then conducted a second proof‐of‐concept test on RNA‐editing changes in *ndhB* and *ndhD*, and splicing variations of *ndhB* in these new tissues. RT‐PCR detected multiple *ndhB* products across WT_RL, *g_*RL, and *sil_*RL, but only *sil_*RL, similar to *P1_12*, yielded a distinct band larger than the expected 1.4‐kb *ndhB* amplicon (Figures 2b and 4c). RT‐PCR of the *ACT2* gene from the cDNAs of *g_*RL and *sil_*RL produced only a band smaller than that from WT genomic DNA, confirming the absence of genomic DNA contamination (Figure 4d).

Using the same TIP sequencing pipeline as illustrated in Figure 1, I found no RNA‐editing changes in either *ndhB* or *ndhD* transcripts, or splicing variations in *ndhB*, between 7‐day‐old seedlings (WT_SDs) and 4‐week‐old adult RLs (WT_RLs) in WT, despite the fact that the expression of *MORF2* in WT_RLs is only about one‐third of that in WT‐SDs (Figure 4e). This finding further supports a dose (threshold)‐dependent impact of *MORF2* on RNA editing. In contrast, consistent with previous studies (Takenaka, Zehrmann, Verbitskiy, Kugelmann, et al. 2012; Bentolila, Oh, et al. 2013; Zhao et al. 2019; Yapa et al. 2023; Tang et al. 2024), I discovered significant RNA‐editing changes at most sites of both *ndhB* and *ndhD* upon *MORF2* overexpression in *g*_RLs and suppression in *sil*_RLs: six out of nine *ndhB* sites and four out of five *ndhD* sites in *g*_RLs, and eight out of nine *ndhB* sites and four out of five *ndhD* sites in *sil*_RLs. I also detected 2.3% ± 1.0% and 6.3% ± 2.1% unspliced *ndhB* variants in *g*_RLs and *sil*_RLs, respectively, which were significantly higher than 0.6% ± 0.4% unspliced *ndhB* variants in WT_RLs (Table 2).

Collectively, this second batch of TIP sequencing analysis demonstrated that chloroplast RNA editing is buffered against moderate changes in *MORF2*, but once *MORF2* levels cross certain thresholds, the editing efficiencies are altered. The consistent discoveries of TIP sequencing in *ndhB* and *ndhD* editing efficiency analysis with previous data further highlight its accuracy and scalability.

## 3 Discussion

Despite the well‐recognized biological importance of RNA editing in plant organelles, quantifying editing efficiencies with high accuracy and scalability remains technically challenging (Tang et al. 2024). Traditional Sanger sequencing approaches, although simple and accessible, lack the resolution and reproducibility necessary for quantitative comparisons across multiple sites and conditions. Meanwhile, advanced next‐generation sequencing (NGS) strategies, such as targeted amplicon‐seq and rRNA‐depleted lncRNA‐seq, require complex workflows, substantial sequencing depth, and high costs, making them impractical for routine applications, particularly for targeted gene analysis or research conducted without access to extensive sequencing infrastructure.

To address these methodological limitations, I developed a rapid, cost‐effective, and reproducible long‐read sequencing approach, termed TIP sequencing, *performed by Plasmidsaurus using Oxford Nanopore Technology*, for digital quantification of RNA editing and transcript maturation at targeted organellar genes. By combining indexed high‐fidelity PCR amplicons with nanopore sequencing and custom in‐house bioinformatic pipelines, this method enables direct and absolute quantification of C‐to‐U editing events with single‐molecule resolution across multiple editing sites. Importantly, the protocol avoids labor‐intensive gel purification, fragmentation, or adapter ligation steps, thus offering significant time and cost advantages over conventional NGS methods.

Application of TIP sequencing to *ndhB* and *ndhD*, two chloroplast transcripts containing numerous editing sites, demonstrated robust read coverage, high reproducibility across biological replicates, and substantial cost savings. The editing efficiency data obtained with this method not only recapitulated previous findings based on Sanger sequencing (Yapa et al. 2023), but also extended them by resolving quantitative differences in a *MORF2* dosage‐dependent manner (Figures 2a and 3). Notably, I observed significant reductions in editing efficiency at six *ndhB* and two *ndhD* sites in the strongly repressed iCRISPRi line *P1–12*, while only modest or no changes were detected in the mildly repressed line *P1–10* and the *KRAB* control.

In a second batch of TIP sequencing analysis on adult rosette leaves, I further tested the scope and reproducibility of the platform. These experiments showed that both upregulation and downregulation of *MORF2* by overexpression of *MORF2‐YFP* or by co‐suppression silencing, respectively, altered RNA editing, whereas developmental downregulation of *MORF2* did not (Figure 4). Together with the analysis from iCRISPRi lines (Figures 2 and 3), these findings suggest that chloroplast RNA editing is buffered against moderate changes in *MORF2* expression but shifts once a threshold of *MORF2* perturbation is crossed. This threshold‐dependent behavior reinforces the central role of *MORF2* as a dosage‐sensitive regulator of plastid RNA editing and highlights the capacity of TIP sequencing to capture subtle yet functionally relevant differences. Additionally, the practice of indexed multiplexing, which combines three replicates and two genes into one TIP sequencing reaction, reduced the cost to only $30 per reaction (i.e., $10 per replicate), dramatically lowering the cost compared with lncRNA‐Seq, which typically averages around $300 per replicate.

Beyond RNA editing, TIP sequencing also uncovered a striking accumulation of intron‐retaining *ndhB* transcripts in Dex‐treated *iCRISPRi‐MORF2* lines and in adult rosette leaves with *MORF2‐YFP* overexpression or co‐suppression. These events cannot be resolved by Sanger sequencing. The increased frequency of reads containing 650–700 bp insertions, corresponding to the unspliced group II intron, directly correlated with the extent of *MORF2* suppression (Figures 2a and 4b; Table 2). These results not only validate the presence of intron‐containing isoforms observed by gel electrophoresis (Figures 2b and 4c), but also provide direct molecular evidence supporting the proposed role of *MORF2* in coordinating RNA maturation and splicing. The observed intron retention phenotype upon *MORF2* perturbation expands its known molecular functions in chloroplast RNA processing, which is likely attributable to its holdase chaperon activity for broad involvement in chloroplast processes, including RNA editing (Takenaka, Zehrmann, Verbitskiy, Kugelmann, et al. 2012), splicing (this study), tetrapyrrole biosynthesis (Yuan et al. 2022), and retrograde signaling (Yapa et al. 2023). This discovery of *MORF2*'s involvement in *ndhB* group II intron splicing further underscores the utility of TIP sequencing in resolving transcript isoforms and splicing intermediates at the single‐molecule level.

Previous research using full‐length transcriptome nanopore sequencing reported that ~32% of *ndhB* transcripts remained unspliced in 5‐week‐old Arabidopsis leaves (Guilcher et al. 2021), a value much higher than the intron‐retention frequencies detected by TIP sequencing in this study (~0.6%–7.8%). This discrepancy likely reflects both methodological and biological factors. Guilcher et al. (2021) employed SMART‐based cDNA synthesis from rRNA‐depleted total RNA, which captures both spliced and incompletely spliced chloroplast transcripts and tends to preserve longer, partially processed RNAs. In contrast, TIP sequencing uses gene‐specific primers to amplify the full coding regions of *ndhB* and *ndhD*. In this setup, PCR tends to favor the shorter, spliced products over the longer intron‐retaining intermediates, leading to an underrepresentation of unspliced variants. Thus, the former method may inflate the proportion of unspliced isoforms, whereas TIP sequencing underestimates them. In addition, *MORF2* expression is developmentally downregulated (Figure 4b). It is possible that in the 5‐week‐old leaves used by Guilcher et al. (2021), *MORF2* expression dropped below a functional threshold, leading to stronger impacts on RNA processing and greater accumulation of unspliced variants. Importantly, Guilcher et al. (2021) analyzed only a single biological sample, likely due to the complex and expensive workflow, limiting the statistical power of their estimates. By contrast, TIP sequencing is simple, cost‐effective, and highly replicable across biological replicates, providing stronger statistical confidence for cross‐genotype and cross‐tissue comparisons. Taken together, these comparisons suggest that the absolute values of unspliced variants or RNA‐editing efficiencies are highly method‐dependent, whereas the reproducibility of TIP sequencing across biological replicates provides the statistical confidence essential for revealing biologically meaningful differences among genotypes and developmental stages.

Another key innovation of this study lies in the suite of bioinformatic tools and scripts I developed to streamline every stage of the data processing workflow, from barcode demultiplexing and strand reorientation to pseudo‐genome alignment and custom intron retention analysis (Figure 1, Methods S1–S4). These bioinformatics tools, written in Perl and Python, are modular, lightweight, and optimized for full‐length amplicon data from Oxford Nanopore platforms. By eliminating the need for large‐scale RNA‐seq pipelines and complex annotation‐dependent software, the open‐source scripts empower other laboratories to adopt long‐read digital RNA editing and splicing variant analysis with minimal computational overhead. To facilitate use, I have included a step‐by‐step usage guide alongside the raw FASTQ files and scripts in a GitHub repository (https://github.com/hualab/Organelle_RNA_sequencing_analysis). Users can simply substitute their own FASTQ files and barcode sequences to analyze their own datasets. I anticipate that these resources will be broadly useful for studying plastid and mitochondrial RNA maturation, RNA‐editing factors, and stress‐responsive transcript isoform dynamics across diverse plant species, and potentially even in other eukaryotic systems, given the widespread presence of RNA editing. In addition, this method can be adapted to analyze splicing variants of nuclear genes in a scalable and cost‐effective manner by multiplexing RT‐PCR amplicons. For example, increasing the number of indexed primers allows more replicates or samples to be included in a single sequencing reaction, further reducing time and cost without significantly compromising data quality. Hence, TIP sequencing is particularly well suited for cross‐genotype or treatment comparisons by focusing on target (marker) genes, where full‐transcriptome RNA‐seq would be impractical or cost‐prohibitive.

In summary, TIP sequencing represents a versatile and accessible platform for targeted analysis of RNA editing and processing in plant organelles. Its key strengths include simplicity, affordability, single‐molecule precision, robust reproducibility, and scalable throughput across multiple genotypes or treatment conditions. By coupling a streamlined experimental workflow with open‐access analytical tools, this method lowers the barrier to entry for plant biologists interested in RNA metabolism and supports a wide range of applications from basic research to translational crop biotechnology.

## 4 Experimental Procedures

### 4.1 Plant Materials, Growth, and Treatments

The iCRISPRi transgenic lines targeting *MORF2* were developed as described in our previous study (Yapa et al. 2023). Briefly, the dCas9‐KRAB fusion protein was expressed under a Dex‐inducible promoter (Aoyama and Chua 1997), while the sgRNA targeting the TSS of *MORF2* was driven by the Arabidopsis *U6–26* promoter (Xing et al. 2014). Four genotypes were used in this study, including the WT and *KRAB* controls (expressing Dex‐inducible *dCas9‐KRAB* only) and two independent iCRISPRi lines (*P1–10* and *P1–12*). Seedlings were grown on half‐strength Murashige and Skoog (1/2 MS) plates supplemented with 5‐μM Dex under long‐day (LD) photoperiod conditions (16 h light at 125 μmol m^−2^ s^−1^, 21°C; 8 h dark, 19°C). Seven‐day‐old seedlings were harvested 2–3 h after dawn for RNA extraction.

Stable *35S:MORF2‐YFP* transgenic plants in the Col‐0 background were also generated in our previous study (Yapa et al. 2023). Synchronized transgenic seeds and Col‐0 WT were grown under LD conditions on a soil mix (1/3 vermiculite, 1/3 peat moss, 1/3 compost soil) for 4 weeks before harvesting adult rosette leaves 2–3 h after dawn for RNA extraction.

### 4.2 RNA Extraction, Reverse Transcription, and PCR Amplification

Total RNA was extracted using the NucleoSpin RNA Plus kit (Macherey‐Nagel) according to the manufacturer's protocol, followed by DNase I treatment (Thermo Fisher Scientific) to remove residual genomic DNA. For reverse transcription, reactions were performed either with 0.5 μg of oligo (dT)25 (IDT) plus random hexamer primers (Thermo Fisher Scientific) using SuperScript III (Thermo Fisher Scientific), or directly with qScript Ultra SuperMix (QuantaBio), to capture full‐length transcripts of *ndhB* and *ndhD*. PCR amplification was performed in a 50‐μL reaction using gene‐specific primers flanking the full‐length coding regions of *ndhB* and *ndhD*, with Phusion High‐Fidelity DNA Polymerase (New England Biolabs) under standard cycling conditions. PCR amplicons were visualized on 1% agarose gels stained with ethidium bromide and quantified using a Qubit Flex Fluorometer with the dsDNA Broad Range (BR) assay kit (Thermo Fisher Scientific).

### 4.3 PCR Amplicon Pooling and Nanopore Sequencing

Forward primers for *ndhB* and *ndhD* PCR products were indexed with unique barcodes for each of the three biological replicates (Tables S2 and S4). PCR products were cleaned using the NucleoSpin Gel and PCR Clean‐up Kit (Macherey‐Nagel) and pooled by genotype. Equimolar mixtures of *ndhB* and *ndhD* amplicons were prepared based on DNA concentrations and product lengths to ensure unbiased representation in the sequencing library (Table S1). Each pooled sample was prepared for nanopore sequencing using the Q20 + Kit V14 chemistry (Oxford Nanopore Technologies) and sequenced on a MinION flow cell (R10.4.1 chemistry) through a commercial service (Plasmidsaurus).

### 4.4 Raw Data Processing and Barcode Demultiplexing

Raw FASTQ reads were basecalled and demultiplexed using Guppy (Plasmidsaurus). To ensure consistent strand orientation, a custom Perl script was developed using *Bio::SeqIO*, *Bio::Seq*, and *Bio::Seq::Quality* modules to reorient each read to the sense strand of *ndhB* and *ndhD* (Method S1). Based on unique 8‐nt barcodes in the forward PCR primers, strand‐corrected FASTQ files representing three biological replicates were reconstructed for each genotype or tissue.

### 4.5 Alignment to Pseudo‐Genome and C‐to‐U Editing Quantification

A custom pseudo‐genome was constructed containing six synthetic chromosomes corresponding to the indexed *ndhB* (chr1–3) and *ndhD* (chr4–6) sequences (Appendix S2). The strand‐corrected FASTQ files reconstructed as above were aligned to the pseudo‐genome using *minimap2* (v2.24) with the following parameters for full‐length cDNA amplicon alignment: *‐ax map‐ont* (Method S2). The resulting SAM files were converted to coordinate‐sorted BAM files using *samtools*. The Integrated Genomics Viewer (IGV, v2.16.0) was used to visualize alignments for manually counting cytidine (C) and thymidine (T) nucleotides at known RNA‐editing sites. Editing efficiency was calculated as *T/*(*C + T*) × 100% for each site.

### 4.6 Insert Size Profiling and Unspliced Transcript Quantification

To identify unprocessed *ndhB* transcripts retaining the group II intron, a Python script was developed to parse CIGAR strings from aligned BAM files for extracting insertions with lengths from 650 to 700 bp (Method S3). Reads containing such large insertions in chr1, chr2, or chr3 (representing the three indexed *ndhB* sequences in the pseudo‐genome) were counted and normalized to the total number of reads mapped to the respective chromosome. The frequency of unspliced *ndhB* transcripts was defined as the proportion of total reads with a 650–700 bp insertion.

### 4.7 Extraction and Local Sequence Alignment of Inserted Introns

Reads containing large insertions were cross‐referenced to the raw FASTQ files to retrieve their full insert sequences, which were saved into separate FASTA files grouped by genotype. A Perl pipeline was used to conduct local sequence alignments between these insert sequences and the *ndhB* group II intron sequence using *BLASTN* with default parameters (Method S4). The sequence similarities and alignment lengths were extracted and analyzed.

### 4.8 Statistical Analysis

Editing efficiencies and intron retention frequencies were compared among genotypes using Student's *t* test. Statistical significance was defined as *p* < 0.05. For multiple replicates, means and standard deviations (SD) are reported.

### 4.9 Use of Artificial Intelligence Tools

ChatGPT (OpenAI) was used during manuscript preparation to assist with language editing and to refine custom Perl and Python scripts for data analysis. All scientific content, including experimental design, data interpretation, and final code implementation, was independently developed, verified, and approved by the author. No AI‐generated content was used to create or alter research data or conclusions.

## Author Contributions

Z.H. conceived the project, designed the research, performed the experiments, analyzed the data, and wrote the paper.

## Conflicts of Interest

The author declares no conflicts of interest.

## Supporting information


**Appendix S1:** Raw FASTQ files generated from TIP sequencing of iCRISPRi lines.

**Appendix S2:** Sequence of the synthetic pseudo‐genome used for read alignment.

**Appendix S3:** Reference sequence of the group II intron from the *ndhB* gene.

**Figure S1:** Sanger sequencing analysis of the first RNA editing site in *ndhB* transcripts from *iCRISPRi‐MORF2* line *P1–12*.

**Data S1** **Method S1:** Perl script “filter_barcoded_fastq_seqs.pl” for converting raw FASTQ reads into a strand‐corrected format.

**Data S2** **Method S2:** Bash script “run_minimap_alignments.sh” for aligning strand‐corrected FASTQ files to the custom pseudo‐genome using minimap2.

**Data S3** **Method S3:** Python script “count_groupII_inserts_and_extract.py” for identifying and extracting intron‐containing *ndhB* transcripts.

**Data S4** **Method S4:** Perl script “local_align_inserts_vs_intron_blast.pl” for BLASTN alignment of intron‐containing *ndhB* transcripts against the reference *ndhB* group II intron sequence.

**Table S1:** Equimolar mixture of *ndhB* and *ndhD* PCR products included in the final pool for TIP sequencing.

**Table S2:** Sequence depth of *ndhB* and *ndhD* from indexed PCR amplicons aligned to each gene‐specific reference sequence across seven genotypes or tissues.

**Table S3:** BLASTN alignment statistics comparing unspliced intron sequences to the reference *ndhB* group II intron.

**Table S4:** Primers used in this study.

## Data Availability

The data supporting the findings of this study are included in the Supporting Information accompanying this article. The complete bioinformatics pipeline, including sequence data (Appendices [Link], [Link]–S3) and analysis scripts (Methods [Link], [Link], [Link]–S4), is also available on GitHub (https://github.com/hua‐lab/Organelle_RNA_sequencing_analysis).
