# Supplementary material for: Rapid and Cost‐Effective Digital Quantification of RNA Editing and Maturation in Organelle Transcripts by Oxford Nanopore Target‐Indexed‐PCR (TIP) Sequencing
Source: Plant Direct. 2025 Oct 20;9(10):e70111. doi: 10.1002/pld3.70111 (PMC12537063; doi:10.1002/pld3.70111)
Supplement: Supplementary file 4 — Figure S1: Sanger sequencing analysis of the first RNA editing site in ndhB transcripts from iCRISPRi‐MORF2 line P1–12. [file PLD3-9-e70111-s006.docx]

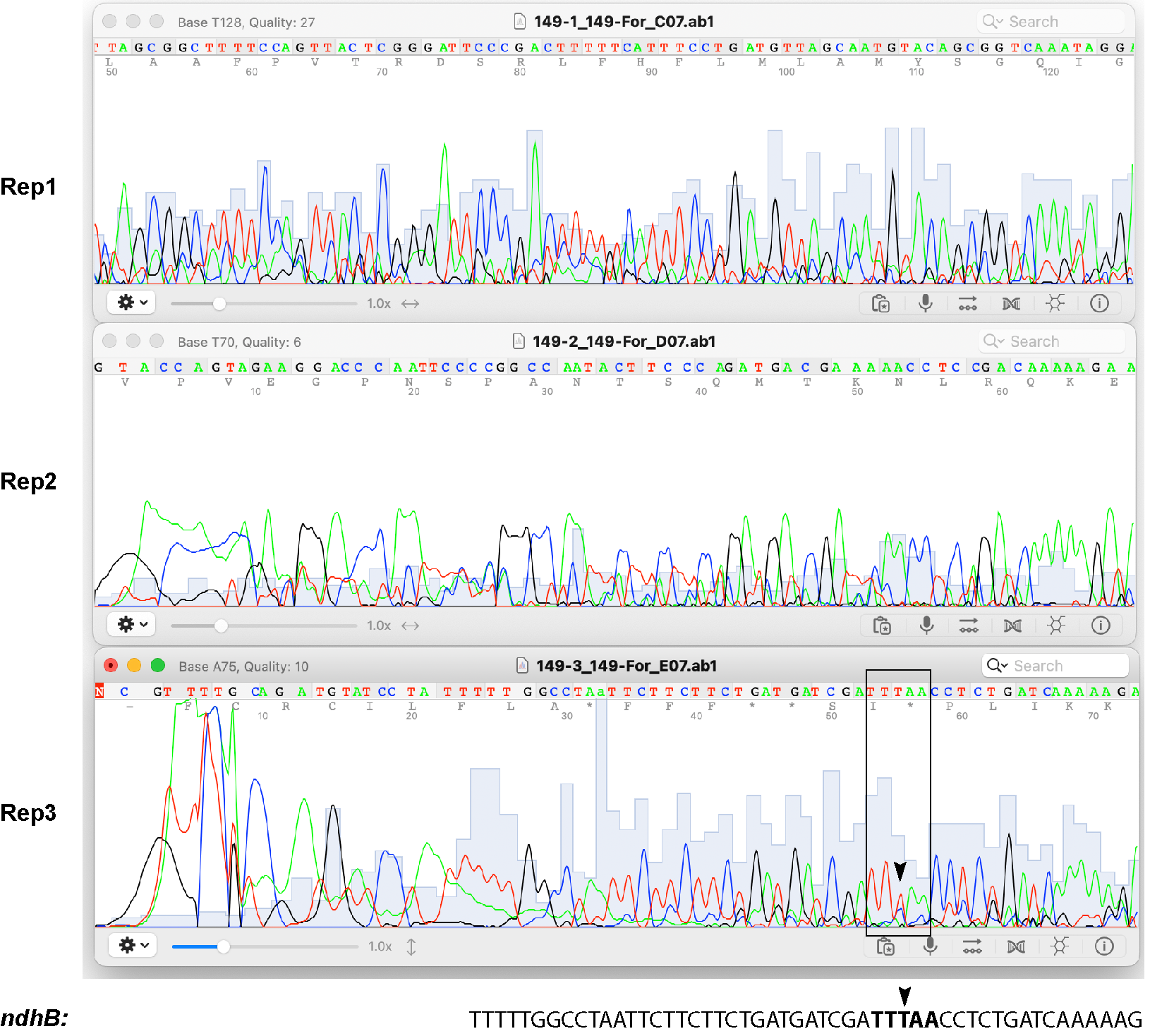


**Fig. S1. Sanger sequencing analysis of the first RNA editing site in *ndhB* transcripts from *iCRISPRi-MORF2* line *P1-12*.**

A 317-bp RT-PCR product encompassing the first *ndhB* C-to-U editing site was gel-extracted and Sanger sequenced from three biological replicates of 7-d-old *iCRISPRi-MORF2* line *P1-12* seedlings treated with 5 μM. Only Replicate 3 yielded a relatively clean chromatogram, allowing visual estimation of editing efficiency based on peak areas of C (blue) and T (red) at the editing site (arrowhead). Chromatograms from the other two replicates were of poor quality, with overlapping peaks across the sequence, precluding accurate quantification. These results illustrate the limited reproducibility and low resolution of Sanger sequencing for quantitative RNA editing analysis. The reference *ndhB* transcript sequence is shown at the bottom of the panel.
