# Supplementary material for: Rapid and Cost‐Effective Digital Quantification of RNA Editing and Maturation in Organelle Transcripts by Oxford Nanopore Target‐Indexed‐PCR (TIP) Sequencing
Source: Plant Direct. 2025 Oct 20;9(10):e70111. doi: 10.1002/pld3.70111 (PMC12537063; doi:10.1002/pld3.70111)
Supplement: Supplementary file 5 — Data S1 Method S1: Perl script “filter_barcoded_fastq_seqs.pl” for converting raw FASTQ reads into a strand‐corrected format. [file PLD3-9-e70111-s004.pdf]

```perl
#!/usr/bin/perl

# Script: filter_barcoded_fastq_seqs.pl
# Tested on: macOS (ARM64) with Perl 5.40.1 and BioPerl ≥ 1.7.8
#
# Description:
#   Converts raw FASTQ reads into strand-corrected format by identifying barcode sequences
#   and reorienting each read to match the sense strand of ndhB or ndhD.
#
# Usage:
#   Save this script as "filter_barcoded_fastq_seqs.pl" in the same directory as the four raw FASTQ files,
#   then run:
#       perl filter_barcoded_fastq_seqs.pl
#
# Dependencies:
#   - Bio::SeqIO
#   - Bio::Seq
#   - Bio::Seq::Quality
#
# Contact:
#   Dr. Zhihua Hua – hua@ohio.edu | ORCID: 0000-0003-1177-1612


use strict;
use warnings;
use Bio::SeqIO;
use Bio::Seq;
use Bio::Seq::Quality;

# Define barcode sequences
my %barcodes = map { $_ => 1 } qw(ATGCTAGC CGTACGTA TACGATCG);

# List of raw FASTQ files
my @input_fastqs = qw(
    45CNXP_1_WT.fastq
    45CNXP_2_Krab.fastq
    45CNXP_3_12-1.fastq
    45CNXP_4_10-1.fastq
);

foreach my $input_fastq (@input_fastqs) {

    # Create input SeqIO stream
    my $in = Bio::SeqIO->new(-file => $input_fastq, -format => 'fastq');

    # Create output SeqIO writers for each barcode
    my %outs;
    for my $bc (keys %barcodes) {
        my $outfile = "${bc}_$input_fastq";
        $outs{$bc} = Bio::SeqIO->new(-file => ">$outfile", -format => 'fastq');
    }

    while (my $seq_obj = $in->next_seq) {
        my $seq  = $seq_obj->seq;
        my $id   = $seq_obj->id;
        my $qual_ref = $seq_obj->qual;
        my @qual_array = @$qual_ref;

        my $rc_obj = $seq_obj->revcom;
        my @rc_qual_array = reverse @qual_array;
        my $matched = 0;

        # Check both sense and reverse-complement sequences
        for my $s_idx (0, 1) {
            my $s_obj  = $s_idx ? $rc_obj : $seq_obj;
            my $s_seq  = $s_obj->seq;
            my $s_qual = $s_idx ? \@rc_qual_array : \@qual_array;
```

```perl
            for my $len (4..8) {
                my $prefix = substr($s_seq, 0, $len);
                if ($barcodes{$prefix}) {
                    my $clean_obj = Bio::Seq::Quality->new(
                        -id   => $id,
                        -seq  => $s_seq,
                        -qual => $s_qual,
                    );
                    $outs{$prefix}->write_seq($clean_obj);
                    $matched = 1;
                    last;
                }
            }
            last if $matched;
        }
    }
}

print "Barcode-based filtering complete. Output written to separate FASTQ files.\n";
```
