# Supplementary material for: Rapid and Cost‐Effective Digital Quantification of RNA Editing and Maturation in Organelle Transcripts by Oxford Nanopore Target‐Indexed‐PCR (TIP) Sequencing
Source: Plant Direct. 2025 Oct 20;9(10):e70111. doi: 10.1002/pld3.70111 (PMC12537063; doi:10.1002/pld3.70111)
Supplement: Supplementary file 6 — Data S2 Method S2: Bash script “run_minimap_alignments.sh” for aligning strand‐corrected FASTQ files to the custom pseudo‐genome using minimap2. [file PLD3-9-e70111-s001.pdf]

```bash
#!/bin/bash

# Script: run_minimap_alignments.sh
# Tested on: macOS (ARM64) with minimap2 v2.29 and samtools v1.21
#
# Description:
#   Aligns strand-corrected FASTQ files to a custom pseudogenome using minimap2,
#   followed by BAM file sorting and indexing using samtools.
#
# Usage:
#   1. Rename "Method S2.txt" as "run_minimap_alignments.sh".
#   2. Save this script in the same directory as:
#        - The strand-corrected FASTQ files
#        - The pseudo-genome file renamed as "pseudo_genome_ndhBD.fa"
#   3. Run the following commands:
#        chmod +x ./run_minimap_alignments.sh
#        ./run_minimap_alignments.sh
#
# Dependencies:
#   - minimap2
#   - samtools
#
# Contact:
#   Dr. Zhihua Hua – hua@ohio.edu | ORCID: 0000-0003-1177-1612


# Set the number of threads
THREADS=4

# Reference genome (pseudo-genome)
REF="pseudo_genome_ndhBD.fa"

# Make sure the reference is indexed for minimap2
if [ ! -f "$REF.mmi" ]; then
    echo "Indexing reference genome..."
    minimap2 -d "$REF.mmi" "$REF"
fi

# Create output directory
mkdir -p minimap_alignments

# List of filtered FASTQ files
FILES=(

TACGATCG_45CNXP_4_10-1.fastq
TACGATCG_45CNXP_3_12-1.fastq
TACGATCG_45CNXP_2_Krab.fastq
TACGATCG_45CNXP_1_WT.fastq
CGTACGTA_45CNXP_4_10-1.fastq
CGTACGTA_45CNXP_3_12-1.fastq
CGTACGTA_45CNXP_2_Krab.fastq
```

```
CGTACGTA_45CNXP_1_WT.fastq
ATGCTAGC_45CNXP_4_10-1.fastq
ATGCTAGC_45CNXP_3_12-1.fastq
ATGCTAGC_45CNXP_2_Krab.fastq
ATGCTAGC_45CNXP_1_WT.fastq

)

# Loop over each FASTQ file and run minimap2
for FILE in "${FILES[@]}"; do
    SAMPLE=$(basename "$FILE" .fastq)
    echo "Aligning $SAMPLE..."

    minimap2 -ax map-ont -t $THREADS "$REF.mmi" "$FILE" | \
        samtools sort -@ $THREADS -o minimap_alignments/${SAMPLE}.sorted.bam

    samtools index minimap_alignments/${SAMPLE}.sorted.bam
    echo "Alignment complete: minimap_alignments/${SAMPLE}.sorted.bam"
done

echo "All alignments completed and indexed."
```
