# Supplementary material for: Rapid and Cost‐Effective Digital Quantification of RNA Editing and Maturation in Organelle Transcripts by Oxford Nanopore Target‐Indexed‐PCR (TIP) Sequencing
Source: Plant Direct. 2025 Oct 20;9(10):e70111. doi: 10.1002/pld3.70111 (PMC12537063; doi:10.1002/pld3.70111)
Supplement: Supplementary file 10 — Table S2: Sequence depth of ndhB and ndhD from indexed PCR amplicons aligned to each gene‐specific reference sequence across seven genotypes or tissues. [file PLD3-9-e70111-s011.docx]

**Table S2**. Sequence depth of *ndhB* and *ndhD* from indexed PCR amplicons aligned to each gene-specific reference sequence across seven genotypes or tissues.

| **Sample** | **Barcode** | ***ndhB*** | ***ndhB* reads** | ***ndhD*** | ***ndhD* reads** |
| --- | --- | --- | --- | --- | --- |
| WT (Replicate 1) | ATGCTAGC | chr1 | 531 | chr4 | 636 |
| *KRAB* (Replicate 1) | ATGCTAGC | chr1 | 614 | chr4 | 535 |
| *P1_12* (Replicate 1) | ATGCTAGC | chr1 | 501 | chr4 | 470 |
| *P1_10* (Replicate 1) | ATGCTAGC | chr1 | 585 | chr4 | 594 |
| WT (Replicate 2) | CGTACGTA | chr2 | 439 | chr5 | 318 |
| *KRAB* (Replicate 2) | CGTACGTA | chr2 | 471 | chr5 | 365 |
| *P1_12* (Replicate 2) | CGTACGTA | chr2 | 330 | chr5 | 251 |
| *P1_10* (Replicate 2) | CGTACGTA | chr2 | 337 | chr5 | 341 |
| WT (Replicate 3) | TACGATCG | chr3 | 196 | chr6 | 258 |
| *KRAB* (Replicate 3) | TACGATCG | chr3 | 215 | chr6 | 271 |
| *P1_12* (Replicate 3) | TACGATCG | chr3 | 122 | chr6 | 170 |
| *P1_10* (Replicate 3) | TACGATCG | chr3 | 186 | chr6 | 255 |
| WT_RL (Replicate 1) | ATGCTAGC | chr1 | 594 | chr4 | 593 |
| *g_*RL (Replicate 1) | ATGCTAGC | chr1 | 756 | chr4 | 541 |
| *sil_*RL (Replicate 1) | ATGCTAGC | chr1 | 787 | chr4 | 630 |
| WT_RL (Replicate 2) | CGTACGTA | chr2 | 270 | chr5 | 173 |
| *g_*RL (Replicate 2) | CGTACGTA | chr2 | 310 | chr5 | 307 |
| *sil_*RL (Replicate 2) | CGTACGTA | chr2 | 233 | chr5 | 273 |
| WT_RL (Replicate 3) | TACGATCG | chr3 | 96 | chr6 | 127 |
| *g_*RL (Replicate 3) | TACGATCG | chr3 | 136 | chr6 | 184 |
| *sil_*RL (Replicate 3) | TACGATCG | chr3 | 70 | chr6 | 95 |
| † The barcoded *ndhB* and *ndhD* reference sequences correspond to chr1-3 and chr4-6, respectively, as organized in a custom pseudo-genome (Appendix S2). | | | | | |
