# Supplementary material for: Rapid and Cost‐Effective Digital Quantification of RNA Editing and Maturation in Organelle Transcripts by Oxford Nanopore Target‐Indexed‐PCR (TIP) Sequencing
Source: Plant Direct. 2025 Oct 20;9(10):e70111. doi: 10.1002/pld3.70111 (PMC12537063; doi:10.1002/pld3.70111)
Supplement: Supplementary file 11 — Table S3: BLASTN alignment statistics comparing unspliced intron sequences to the reference ndhB group II intron. [file PLD3-9-e70111-s012.docx]

**Table S3.** BLASTN alignment statistics comparing unspliced intron sequences to the reference *ndhB* group II intron.

|  | **WT** | **KRAB** | **P1_10** | **P1_12** |
| --- | --- | --- | --- | --- |
| Sequence similarity (%; mean ± sd) | 99.1 ± 0.8 | 98.8 ± 2.4 | 99.1 ± 1.5 | 98.8 ± 2.0 |
| Alignment length (nt; mean ± sd) | 686 ± 2 | 685 ± 5 | 685 ± 4 | 684 ± 6 |
|  | **WT_RL** | ***g*_RL** | ***sil*_RL** | **ALL** |
| Sequence similarity (%; mean ± sd) | 99.1 ± 0.8 | 98.9 ± 1.2 | 99.1 ± 0.9 | 99.0 ± 1.6 |
| Alignment length (nt; mean ± sd) | 680 ± 7 | 683 ± 6 | 684 ± 4 | 684 ± 5 |
