# Supplementary material for: Rapid and Cost‐Effective Digital Quantification of RNA Editing and Maturation in Organelle Transcripts by Oxford Nanopore Target‐Indexed‐PCR (TIP) Sequencing
Source: Plant Direct. 2025 Oct 20;9(10):e70111. doi: 10.1002/pld3.70111 (PMC12537063; doi:10.1002/pld3.70111)
Supplement: Supplementary file 12 — Table S4: Primers used in this study. [file PLD3-9-e70111-s005.docx]

**Table S4.** Primers used in this study.

| **Reactions** | **Primer Name** | **Sequence** |
| --- | --- | --- |
| RT-PCR | *ndhB*1_For | TCATTTGCTTCTCTTCGATGG |
|  | *ndhB*1_Rev | TGTTATAGCCATTTCTGTACATTCAA |
|  | *ndhB*2_3_For | CCAAACGAACAATTTCAACG |
|  | *ndhB*2_3_Rev | AGGGAAAGCTTGAACCCAAT |
|  | *ACT2*_For | GGCATCACACTTTCTACAATGAG |
|  | *ACT2*_Rev | ACCCTCGTAGATTGGCACAG |
| Multiplexing RT-PCR | Rep1_*ndhB*_For | **ATGCTAGC**TTTTTGGCCTAATTCTTCTTCTGA |
|  | Rep2_*ndhB*_For | **CGTACGTA**TTTTTGGCCTAATTCTTCTTCTGA |
|  | Rep3_*ndhB*_For | **TACGATCG**TTTTTGGCCTAATTCTTCTTCTGA |
|  | *ndhB*_Rev | AATCGCAATAATCGGGTTCATT |
|  | Rep1_*ndhD*_For | **ATGCTAGC**AACAACTCGAAGTATGGGTC |
|  | Rep2_*ndhD*_For | **CGTACGTA**AACAACTCGAAGTATGGGTC |
|  | Rep3_*ndhD*_For | **TACGATCG**AACAACTCGAAGTATGGGTC |
|  | *ndhD*_Rev | GGCAATGCAAGGGAAGCC |
| qPCR | *MORF2*_qPCR_For | AGCTAAGCCAGTATGAGGATGA |
|  | *MORF2*_qPCR_Rev | CCAAAGTAAGCCCGGTCCAT |
|  | *YFP_qPCR_For* | ACGGCAGCGTGCAGCTC |
|  | *YFP_qPCR_Rev* | AGCTCGTCCATGCCGAGAG |
|  | *ACT2*_qPCR_For | GGCATCACACTTTCTACAATGAG |
|  | *ACT2*_qPCR_Rev | ACCCTCGTAGATTGGCACAG |
|  | *PP2A*_qPCR_For | AAGCTTGGTGCTCTTTGCAT |
|  | *PP2A*_qPCR_Rev | CCATTACTGGAGCGAGAAGC |
| † The underlined and bolded 8-nucleotide sequences at the 5′ end of the forward primers for *ndhB* and *ndhD* indicate the barcodes used for multiplexed RT-PCR across three biological replicates. | | |
